# The Role of SH2 Domain-containing Leukocyte Phosphoprotein of 76 kDa in the Regulation of Immune Cell Development and Function

**DOI:** 10.4110/in.2009.9.3.75

**Published:** 2009-06-30

**Authors:** Gary A. Koretzky

**Affiliations:** Department of Pathology and Laboratory Medicine and Department of Medicine, Abramson Family Cancer Research Institute, University of Pennsylvania School of Medicine, Philadelphia, PA, 19104, USA.

**Keywords:** SH2 domain-containing leukocyte phosphoprotein of 76 kDa, signal transduction, T cells, integrins, immunoreceptors, adapter proteins

## Abstract

Recent years have seen an explosion of new knowledge defining the molecular events that are critical for development and activation of immune cells. Much of this new information has come from a careful molecular dissection of key signal transduction pathways that are initiated when immune cell receptors are engaged. In addition to the receptors themselves and critical effector molecules, these signaling pathways depend on adapters, proteins that have no intrinsic effector function but serve instead as scaffolds to nucleate multimolecular complexes. This review summarizes some of what has been learned about one such adapter protein, SH2 domain-containing leukocyte phosphoprotein of 76 kDa (SLP-76), and how it regulates and integrates signals after engagement of immunoreceptors and integrins on various immune cell lineages.

In the last twenty-five years, since the initial description of the molecular components of the T cell antigen receptor (TCR), much attention has been focused on understanding the biochemical events that occur when that receptor is engaged by antigen (1). Elegant work by a number of laboratories showed that the most proximal signal that initiates TCR-induced responses is activation of a cascade of protein tyrosine kinases (PTKs)(2,3), first members of the Src family, then the Syk family PTK ζ-associated protein of 70 kDa (Zap-70), followed by activation of members of the Tec family. These PTKs then phosphorylate numerous substrates leading to the stimulation of multiple second messengers. The signal transduction cascades are then integrated in the cell, leading to the appropriate biologic response. As the various signaling pathways were being defined, a series of studies began investigating how this critical integration occurred. This work led to the identification of a number of adapter proteins. These molecules act as scaffolds, which through their modular domains are able to bind a discrete number of other molecules, thus creating and appropriately positioning within the cell complexes of signaling molecules (4). Subsequent studies demonstrated that this model for adapter protein function is not restricted to signaling downstream of the TCR, as these adapters (or closely related molecules) are found in all immune cell lineages and appear essential for supporting the function of multiple immunoreceptors, including the B cell antigen receptor and receptors for various immunoglobulin constant regions. More recently, it has been shown that a subset of these adapters are also critical for signaling downstream of other key immune cell receptors, including costimulatory receptors and various adhesion molecules including integrins. Interestingly, it appears that the molecular rules for how these adapter proteins function overlap but are not identical.

## SRC HOMOLOGY 2 (SH2) DOMAIN-CONTAINING LEUKOCYTE PHOSPHOPROTEIN OF 76 kDa (SLP-76)

SLP-76 was identified originally in a screen designed to discover substrates of the PTKs stimulated by ligation of the TCR (5). The gene encoding SLP-76 had not been previously studied, and upon initial sequence analysis it appeared likely that SLP-76 functioned as an adapter, as it consisted of several modular domains, each with the capability to bind other proteins. By RNA and protein analysis, it was shown that SLP-76 is present in all hematopoietic lineages, except for mature B cells (6). In its N-terminal region, SLP-76 possesses three tyrosine residues (Y112, Y128, and Y145) demonstrated to be substrates of syk family PTKs (ZAP-70 in T cells and Syk in other immune cell types) (7). Each of these tyrosines is in a consensus motif that predicts binding to SH2 domains of other proteins. C-terminal to the phosphorylatable tyrosines is a proline-rich region that is predicted to bind Src homology 3 (SH3) domains of other proteins, and at its most C-terminus, SLP-76 possesses an SH2 domain that is able to bind other proteins, following their own tyrosine phosphorylation.

Work from several laboratories has defined the binding partners for these three SLP-76 domains. Tyrosines 112 and 128 are in the same motif (DYESP) and when phosphorylated create an excellent docking site for the SH2 domains of the guanine nucleotide exchange factor Vav and the adapter protein Nck (8-12). Phosphorylated tyrosine 145, in a DYEPP motif, is an excellent binder of the SH2 domain of Tec family PTKs (13,14). The proline-rich central region of SLP-76 has residues that also bind to Tec family PTKs (SH3 domains of these molecules) as well as residues that interact with phospholipase Cγ (either γ1 or γ2 depending on the cell lineage) (15). The central region of SLP-76 also binds to members of the Grb2 family of adapter proteins, however with a much greater preference for Gads over Grb2 itself (16-18). The C-terminal SLP-76 SH2 domain binds the serine/threonine kinase HPK-1 (19) as well as the adapter protein adhesion and degranulation- promoting adapter protein (ADAP) (20,21).

SLP-76 is found in the cytosol of resting cells. It constitutively binds to its partners that interact via its central proline-rich region, and it inducibly associates with its binders that require tyrosine phosphorylation after receptor engagement (and activation of the receptor-associated PTKs). The ability of SLP-76 to support signaling downstream of both immunoreceptors and integrins appears to require its relocalization from the cytosol to signaling clusters that form near the activated receptors (22,23). Our current understanding of how SLP-76 traffics within cells is described below.

## THE ROLE OF SLP-76 IN T CELL DEVELOPMENT AND PERIPHERAL T CELL ACTIVATION

As an adapter protein, it was initially hypothesized that SLP-76 would serve to increase the efficiency of signaling via cell surface receptors by bringing key components of the signaling pathways together in the right location within the cell. Support for this notion came from initial studies in the Jurkat cell line model, where SLP-76 was overexpressed by gene transfection and shown to increase readouts of activation following engagement of the TCR (24). Subsequent studies making use of Jurkat cells lacking SLP-76 (25) or mice made deficient in the adapter through gene targeting (26,27) revealed the essential role SLP-76 plays in TCR signaling. In both of these model systems, it was shown that in the absence of SLP-76, engagement of the TCR still activates Src and Syk family PTKs; however, all PTK-dependent downstream signals are abrogated. These findings led to the notion that SLP-76, through its basal and inducible interactions with other proteins, nucleates a multimolecular complex that is essential for TCR signal transduction. The *in vivo* consequence of SLP-76 loss in the T cell compartment is a complete block in thymocyte development at the double negative 3 (DN3) stage, the point during which the pre-TCR must signal its successful expression following TCR gene recombination. Failed signaling at this point leads to thymocyte death and the absence of double positive (DP) and single positive (SP) cells. Mice lacking SLP-76 therefore have no T cells in their peripheral lymphoid organs. More recent studies making use of "floxed" SLP-76 alleles demonstrate that if the gene for SLP-76 is not deleted until after DP thymocytes appear, SLP-76 loss in this compartment abrogates selection, leading to thymocyte "death by neglect" (28). Deletion of the SLP-76 gene later on in development (after T cells have matured and populated the periphery) leads to failed T cell activation following antigen/MHC encounter (Maltzman J, Personal Communication).

## CONSEQUENCES OF SLP-76 LOSS IN NON-T CELL COMPARTMENTS

Examination of SLP-76-deficient animals has revealed critical roles for this adapter in multiple other cell lineages, although these cell types appear in normal numbers in SLP-76-null mice. Neutrophils lacking SLP-76 signal poorly through their Fc receptors and cell surface integrins (29). This results in diminished adherence and spreading of neutrophils following integrin engagement and reduced neutrophil degranulation when either Fc receptors or integrins are ligated. Although SLP-76-deficient neutrophils are still able to phagocytose and kill bacteria, lack of this adapter protein results in diminished *in vivo* inflammatory responses. Mast cells similarly rely on SLP-76 for their function, as engagement of the high affinity receptor for IgE fails to initiate the appropriate downstream biochemical signals when SLP-76 is missing. This defect results in diminished cytokine production and failed *in vivo* passive systemic anaphylaxis (30,31).

Another cell type that requires SLP-76 for both immunoreceptor (Fc receptor) and integrin signaling is platelets. Lack of SLP-76 results in failed platelet aggregation or degranulation in response to collagen (which makes use of both integrins and the GPVI receptor that pairs with the common γ chain of Fc receptors) (32). SLP-76-deficient platelets additionally fail to transduce signals when stimulated by fibrinogen via the α2bβ3 receptor (33).

Additional studies of SLP-76-deficient mice revealed another striking phenotype that emerged in the absence of this adapter protein during early development. The initial analysis of SLP-76-deficient mice revealed non-Mendelian inheritance of homozygous null alleles in the adult population. Subsequent analysis demonstrated that SLP-76 deficiency is present in a Mendelian ratio until shortly after birth. Evaluation of fetuses from SLP-76^+/-^ matings gave rise to the observation that it was possible to genotype the SLP-76 deficiency by eye, as fetuses lacking two SLP-76 alleles appeared grossly hemorrhagic. Later, it was shown that what originally appeared as bleeding was in fact the presence of blood-filled lymphatic vessels, as it was learned that loss of SLP-76 prevented normal separation of blood from lymphatic vessels (34). This defect leads to altered hemodynamics of the SLP-76-deficient mice resulting in premature death. The molecular underpinning of this defect and the signaling pathway that is causal remain under investigation (35).

## A MODEL FOR HOW SLP-76 SUPPORTS IMMUNORECEPTOR SIGNALING

Studies from multiple laboratories have resulted in a wealth of data addressing the molecular mechanism by which SLP-76 integrates signals from cell surface receptors. In resting cells, SLP-76 resides in the cytosol, constitutively associated with Gads, a member of the Grb2 family of adapters. When immunoreceptors are engaged, the most proximal biochemical event that occurs is the activation of members of the Src family PTKs. This results in the phosphorylation of the immunoreceptor tyrosine-based activation motifs (ITAMs) present within the CD3 molecules of the TCR on T cells and Fc receptors on other immune cells. The ITAM phosphotyrosines then recruit members of the Syk family PTKs, thus converting the immunoreceptor from an enzymatically inert structure to a receptor with associated PTK activity.

Among the substrates that are rapidly phosphorylated by the activated immunoreceptor complex are adapters including the transmembrane protein linker of activated T cells (LAT) (36). LAT acts as an adapter through its cytoplasmic tyrosines that are phosphorylated once Syk PTKs are recruited to the immunoreceptor ITAMs. Once phosphorylated, the LAT tyrosines are able to bind key signaling molecules including Gads, which, through its association with SLP-76, brings SLP-76 to LAT and into the vicinity of the activated immunoreceptor. Together, LAT and SLP-76 nucleate a large multimolecular complex that includes many of key enzymes and other effector molecules critical for immune cell activation.

Because SLP-76 is also a substrate of Syk family PTKs, activation of immunoreceptors results in SLP-76 tyrosine phosphorylation. This occurs on three residues (Y112, Y128, and Y145) within the N-terminus of SLP-76. Y112 and Y128 are in the appropriate motif to bind to SH2 domains of Vav, a guanine nucleotide exchange factor important for cytoskeletal reorganization, and Nck, another adapter essential for full immune cell activation. Y145 has a binding site for the SH2 domain of Tec family PTKs (ITK in T cells and BTK in other immune cells). These PTKs are critical for initiating calcium and ras signaling in immune cells by activating PLC (γ1 in T cells and γ2 in other immune cells). Initiation of this pathway is facilitated by the interaction of SLP-76 with PLCγ1 and PLCγ2 via the proline-rich central region of SLP-76. PLC localization is further facilitated by the interaction of these enzymes with phospho-LAT. The C-terminal SH2 domain of SLP-76 also recruits key signaling molecules, including the serine/threonine kinase HPK-1. Recent data suggest that HPK-1 can phosphorylate SLP-76 on serine residues (37). The SLP-76 SH2 domain also interacts with another adapter protein, ADAP, once ADAP is phosphorylated by the activated immunoreceptor. ADAP is important for linking the immunoreceptor activation complex with integrins (see below).

Evidence supporting the inducible creation of this multimolecular signaling complex has come from biochemical, imaging, and genetic approaches using cell lines and *in vivo* model systems. Early studies defined the binding partners of LAT and SLP-76, and then mapped residues key for these interactions using cell lines lacking either molecule followed by transfection of wild type or mutant versions of LAT (38) or SLP-76 (16,21,39) for subsequent biochemical analysis.

Interestingly, when transfected SLP-76 or LAT constructs were tagged with fluorochromes and subjected to image analysis, it was found that immunoreceptor engagement resulted in the rapid appearance of microclusters containing the two adapter proteins along with other signaling molecules (22). Subsequent studies following the dynamics of these clusters in real time in living cells demonstrated that the interaction between SLP-76 and LAT via Gads was essential for the normal appearance and movement of these clusters (40).

## TESTING THE MODEL: THE SLP-76/LAT COMPLEX IS CRITICAL FOR IMMUNORECEPTOR SIGNALING

The demonstration that a large multimolecular complex nucleated by LAT and SLP-76 exists did not prove the importance of this complex for immunoreceptor function. SLP-76- and LAT-deficient cell lines and mice were therefore employed to address the causal relationship between the intermolecular interactions, microcluster formation, and immunoreceptor function. The paradigm for the cell line approach was to express wild type or mutant SLP-76 or LAT variants in Jurkat cells lacking these proteins (J14 for SLP-76 and J.CaM2 for LAT) by transfection of cDNAs. For both J14 and J.CaM2, transfection of cDNA encoding a wild type version of the missing molecule completely restored signaling mediated via the TCR. Subsequent studies demonstrated that mutants of SLP-76 or LAT that could not bind to Gads failed to support TCR-mediated activation of the PLC signaling cascade (23,41-43). Complementary studies were then performed in primary murine T cells in which a small peptide that blocked the interaction between SLP-76 and Gads was used and found to markedly interfere with TCR signaling (44).

The *in vivo* importance of the SLP-76/Gads/LAT axis was then demonstrated through the use of transgenic animals expressing SLP-76 mutants on the SLP-76-deficient background and through retroviral transduction of SLP-76 null bone marrow with wild type or mutant variants of SLP-76 followed by transplant into Rag^-/-^ recipients (32,45-47). In both cases, although the phenotype is not as severe as that seen with the complete absence of SLP-76, expression of a mutant SLP-76 molecule that cannot bind to Gads fails to restore normal T cell development. Thus, while SLP-76 deficiency results in a complete block in thymocyte development at the DN3 stage, reconstitution of SLP-76-deficient mice with a mutant of the adapter that cannot bind Gads allows for the development of some DP cells. Few SP thymocytes or peripheral T cells emerge, however, in these mice, and those cells that do develop fail to respond to TCR engagement. Thus, it appears that some TCR function can occur when SLP-76 cannot be localized to LAT via Gads; however, this function is markedly curtailed. Whether the residual function is due to an alternative means to relocalize SLP-76 or instead due to SLP-76 functions elsewhere in the cell is under investigation.

Generating mice in which SLP-76 is altered so that it no longer binds Gads provided the opportunity to investigate the importance of the SLP-76/Gads/LAT axis for signaling via immunoreceptors in non-T cell lineages and for testing the importance of these intermolecular interactions downstream of integrins. Mast cells from mice reconstituted with a SLP-76 mutant that cannot bind Gads signal poorly via their high affinity receptor for IgE (40,48), and platelets expressing the Gads binding mutant in the absence of wild type SLP-76 do not respond to collagen via GPVI (32). However, in all lineages tested, although SLP-76 is critical for responses to cell surface integrins, this SLP-76 function appears to be independent of the Gads-binding region of the adapter (46). This finding along with studies suggesting that LAT is dispensable for integrin signaling indicate that the molecular complexes nucleated by SLP-76 differ between these signaling pathways.

## THE ROLE OF THE SLP-76 SH2 DOMAIN IN SIGNALING FROM IMMUNORECEPTORS TO INTEGRINS

One important consequence of immunoreceptor signaling is to upregulate the avidity of integrins for their ligands (a process known as "inside-out" signaling), thus making immune cells better able to respond to integrin activation (49). Several laboratories have investigated the molecular basis and, in particular, the domains of SLP-76 that are most critical for inside-out signaling. This work led to the finding that the SLP-76 SH2 domain, while dispensable for coupling the TCR to a number of its second messenger cascades (for example, the PLC pathway), appears critical for inside-out signaling. A molecular explanation for this finding was provided by an analysis of ADAP, one of the molecules that interacts with the SLP-76 SH2 domain.

ADAP becomes tyrosine phosphorylated following immunoreceptor engagement by Fyn, one of the Src family PTKs that is inducibly activated upon TCR engagement (20). In fact, ADAP can also associate with Fyn in a large molecular complex. Dissection of this Fyn-associated complex led to the discovery of another adapter molecule designated SKAP55 (Src kinase-associated adapter protein of 55 kDa) (50). SKAP55 binds constitutively to a proline-rich region of ADAP via the SKAP55 SH3 domain (51). Interestingly, it appears that not only do ADAP and SKAP55 associate with each other but that this interaction also is critical for stabilizing ADAP expression, as loss of SKAP55 results in rapid degradation of ADAP in the cell (52).

After the discovery of ADAP and SKAP55, a series of studies were initiated to probe their importance in immune cell signaling. Several groups found that cells lacking these adapter proteins failed to adhere tightly to integrin coated surfaces (53-55). This was found to be due to defects in both inside-out signaling from immunoreceptors to integrins (thus decreasing integrin avidity for ligand) and also to diminished outside-in signaling from integrins into the cell. These observations, however, did not address how SLP-76, ADAP, and SKAP55 were able to intersect with integrins within the cell. A candidate for linking ADAP and SKAP55 to integrins came with the discovery of yet another adapter molecule known as RIAM (Rap1-GTP-interacting adapter molecule) (56). Interest in RIAM as a potential linker was piqued by the finding that RIAM associates with the active form of the small molecular weight GTPase Rap1 (57). Several earlier studies had shown that active Rap1 played an essential role in inside-out signaling to integrins (58-61). How these pieces might all fit together was suggested by the finding that RIAM also binds to SKAP55 (62). Collectively, these studies result in a model suggesting that stimulation of immunoreceptors results in another large SLP-76-nucleated complex, this one centered around the SLP-76 SH2 domain containing ADAP, SKAP55, RIAM, and activated Rap1. It is this complex that is essential for linking immunoreceptors to upregulation of integrin functions. It remains to be determined exactly how this complex is recruited to integrins and whether this complex is built around a SLP-76 molecule that also binds Gads, PLC, and LAT, or if the two signaling scaffolds are distinct from each other.

## EXPLORING THE IMPORTANCE OF THE SLP-76 TYROSINES USING GENOMIC KNOCKIN MICE

The first *in vivo* structure/function studies of SLP-76 making use of mice transgenic for mutant forms of SLP-76 crossed to SLP-76-deficient animals demonstrated that among the three SLP-76 protein interacting domains, the N-terminal tyrosines were most critical for function (45,47). This was shown using a transgene in which the three N-terminal tyrosines were all altered to phenylalanine (Y3F). Although not an exact phenocopy of the SLP-76-null mice, each lineage examined expressing the Y3F variant of SLP76 signaled poorly via immunoreceptors and integrins. Subsequent studies made use of the J14 cell line by transfecting constructs encoding wild type SLP-76 or mutant variants of SLP-76 where one, two, or all three tyrosines were altered to phenylalanine (63). Every combination was studied leading to the observation that Y145 seemed most critical for TCR function and that the combination of Y112 and Y128 altered to phenylalanine seemed to have the next most significant effect. Thus, in J14 cells expressing either the Y145F or Y112/128F mutant, engagement of the TCR couples poorly with the downstream signaling pathways described above. These data as well as the fact that Y112 and Y128 are found in the same DYESP motif while Y145 is present within a slightly different DYEPP motif led us to generate genomic knockin mice in which Y145 or both Y112 and Y128 of SLP-76 were altered to phenylalanine. These mice made it possible to explore the *in vivo* consequences of these mutations (64,65).

The first observation made with the knockin animals was that the perinatal lethality found in SLP-76-null animals is rescued. This finding suggests that although immunoreceptor and integrin signaling (see below) are severely compromised in these mice, sufficient signaling is supported by the SLP-76 mutants to prevent the vascular abnormalities from occurring. This observation provides an important clue that is being pursued to understand what cell type and which receptor on that cell requires SLP-76 to support normal vascular development.

Given the poor signaling via the TCR in the Jurkat system, we were surprised to discover that gross thymic development in the Y145F and Y112/128F mice was remarkably intact in terms of overall cellularity and examination of subsets by CD4/CD8 staining. It was evident, however, through staining for CD5 and surface TCR levels that positive selection was likely aberrant. This finding was extended by evaluating TCR Vβ family usage with the observation that in strains that should have eliminated particular TCR families (due to endogenous superantigen expression), deletion was defective (worse in the Y145F compared to the Y112/128F mice). Thus, it appeared that both positive and negative selection may be impacted by altering the SLP-76 tyrosines. This was confirmed by breeding the knockin mice to animals expressing TCR transgenes that should either signal deletion or positive selection. In both circumstances we found substantial defects in selection, again with the Y145F mice demonstrating a more pronounced phenotype than that observed in the Y112/128F animals. Aberrant selection in the thymi of these mice correlated with failed TCR signaling as expected from the studies of the reconstituted J14 mice.

Generating knockin mice with complementary tyrosine mutations gave us the opportunity to test the hypothesis that two SLP-76 mutants, each conferring a significant phenotype, could complement each other in *trans* in a single cell. To approach this question, we bred the Y145F mice to mice expressing Y112/128F SLP-76 resulting in animals with no wild type SLP-76 alleles but instead one Y145F allele and one Y112/128F encoding chromosome. We were surprised that in every assay of TCR function or thymic development, we found a rescue to nearly wild type levels. Thus, it appears that the two SLP-76 mutants can cooperate with each other when expressed in the same cell.

The knockin mice gave us the opportunity also to investigate the importance of the SLP-76 tyrosines in other lineages. Similar to what we discovered in the T cell compartment, platelets expressing either the Y145F or Y112/128F SLP-76 mutants revealed marked signaling abnormalities downstream of the GPVI receptor (65). Interestingly, while there were defects in integrin signaling in platelets from both knockin strains, the abnormalities appeared greater in the Y112/128F cells compared to their Y145F counterparts. Because the Y to F knockin mice did not demonstrate a vascular phenotype, we were able to investigate the importance of the tyrosines for *in vivo* platelet function. For these studies, we applied an irritant (ferric chloride) to the external carotid artery of mice and then measured blood flow in the artery by Doppler. In wild type mice, the irritated artery stimulated a rapid platelet response resulting in occlusion of the vessel by a stable thrombus. The vast majority of SLP-76 Y145F mice failed entirely to occlude the vessel, and in all but one mouse in which a thrombus formed, the clot that did occur was unstable. The phenotype of the Y112/128F mice tended towards defective thrombus formation but was not nearly as pronounced as seen in the Y145F animals. Based on our studies in T cells, we predicted that the two tyrosine mutant alleles would complement each other in *trans* in the platelet compartment. We were surprised to discover that this was not the case, an observation that we are continuing to pursue.

## SUMMARY

Recent years have seen an explosion of new knowledge informing the scientific community regarding the process by which signals are transmitted from engaged receptors on the surface of cells. Immune cells have proven to be an outstanding model system, as they are easily accessible and can be manipulated both *in vitro* and *in vivo*. Signals initiated by the binding of immunoreceptors and integrins results in the assembly of large multimolecular complexes that are critical for integration of the myriad of second messenger cascades. These complexes are nucleated by key adapter proteins. Interestingly, while some of the rules for the generation and function of these complexes are the same, the molecular details of how they act appear to differ between cell types and receptor systems. Additional information about how these complexes regulate key biologic outcomes will provide useful clues as attempts are made to manipulate signaling events for therapeutic benefit.
